# Pallister–Hall syndrome with orofacial narrowing and tethered cord: a case report

**DOI:** 10.1186/s13256-018-1868-8

**Published:** 2018-11-29

**Authors:** Femia Hayek

**Affiliations:** Department of Pediatrics, Rafic Hariri University Hospital, Bir-Hassan, Jinah, Beirut, Lebanon

**Keywords:** Pallister–Hall syndrome, Hypothalamic hamartoma, Tethered cord

## Abstract

**Background:**

Pallister–Hall syndrome is a rare, autosomal dominant, genetic disorder characterized by different congenital abnormalities: hypothalamic hamartoblastoma, bifid or shortened epiglottis, polydactyly, renal anomalies, and imperforate anus.

**Case presentation:**

In this case report, we describe the case of a 13-year-old Lebanese-Armenian boy born with Pallister–Hall syndrome showing newly associated manifestations (orofacial narrowing and tethered cord), and currently showing a spontaneous puberty with normal growth pattern following management with growth hormones.

**Conclusions:**

This case report shows a practical approach to this very rare syndrome, mainly with testosterone and growth hormones, and its follow-up in the long term. Being familiar with such cases may allow improvement of our knowledge for better management in the future.

## Background

Pallister–Hall syndrome (PHS) is a rare, autosomal dominant, genetic condition [1]. Familial cases with an autosomal dominant inheritance pattern have been reported [2, 3]. This complex syndrome involves a mutation in the *GLI3* gene, which is located on the short arm of chromosome 7 [1]. The importance of this gene is that it regulates downstream genes of the hedgehog pathway important to the formation of the neural tube, craniofacial structures, otic vesicles, and distal limb buds [1]. PHS is characterized by different congenital abnormalities: hypothalamic hamartoblastoma, bifid or shortened epiglottis, polysyndactyly (more than five fingers per hand), renal anomalies, and imperforate anus [4, 5]. Hypothalamic hamartoblastoma is characterized by a protrusion of the third cerebral ventricle, displacement of the optic tract, interruption of the pituitary stalk, and hypoplasia of the pituitary gland. Other reported abnormalities associated with PHS include colonic aganglionosis [6], epilepsy [7], hypopituitarism [8], congenital cardiac defects [9], and adrenal abnormalities [10]. We report the case of a male patient with PHS showing new associated features.

## Case presentation

**Fig. 1:**
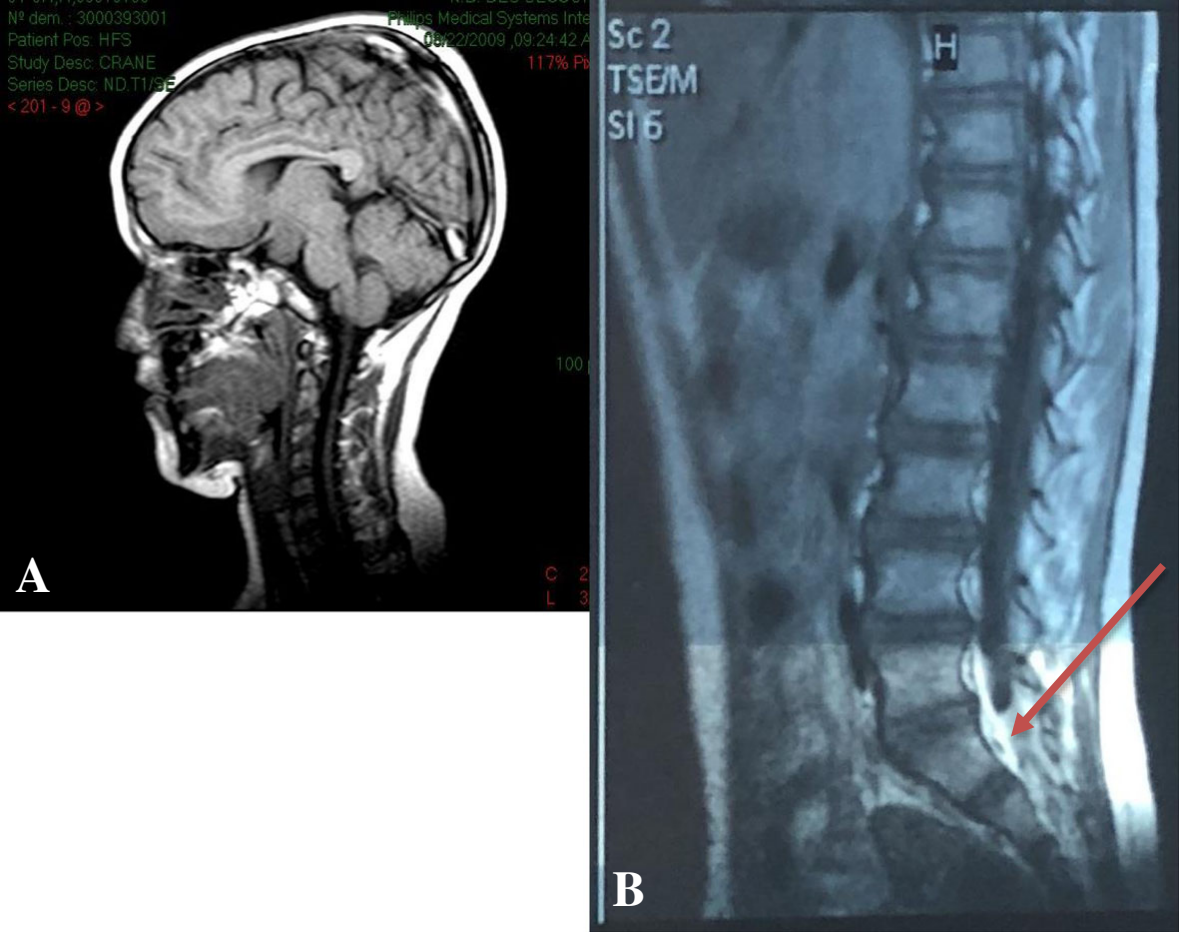
Magnetic resonance imaging of the brain and the spine. **a** Magnetic resonance imaging of the brain – hypothalamic hamartoma. **b** Magnetic resonance imaging of the spine (T2-weighted scan) – fibrolipoma of the filum (*red arrow*)

Our patient is a 13-year-old Lebanese-Armenian boy born in March 2004 from non-consanguineous and healthy parents, and after normal pregnancy and delivery. On neonatal examination, he was found to have hypertelorism, broad nasal bridge, choanal atresia by failing to pass nasal tube, heart murmur uncovering an interventricular communication, polysyndactyly, anal stenosis, micropenis (length, 1 cm; normal length for the age, 2–4.5 cm), and cryptorchidism. Because of the midline structural abnormalities, a magnetic resonance imaging (MRI) of his brain was performed and showed a hypothalamic hamartoma which was the key for the diagnosis of PHS (Fig. 1a).

At the age of 3 days, he was operated on for his choanal atresia. This operation revealed a narrow esophageal opening necessitating the use of smashed food late in his pediatric age. Imaging results, including upper gastrointestinal series and barium enema, confirmed the esophageal narrowing and revealed anal stenosis for which he was operated on at the age of 6 months. However, he remained severely constipated despite the surgical and medical treatment. During the same period, he developed seizures and was controlled by anticonvulsive treatment despite a normal electroencephalogram (EEG).

Regarding congenital limb malformations, he showed dysplastic nails with brachydactyly and post-axial polydactyly of his right hand. He also had broad great toes with syndactyly of the right fourth and fifth metatarsi. These malformations were managed subsequently.

At the age of 4 years, he started complaining of abnormal gait and an inability to keep up with peers during playing. A T2-weighted MRI sequence of his spine showed a tethered cord at L3 level by a fibrolipoma of the filum (thickness, 4 cm) (Fig. 1b), which was immediately and surgically removed.

Meanwhile, and because of bad school performance, a work-up was done including electric conductance of the acoustic nerve showing absent conductance on the right side and 20% activity on the left side. His condition was ameliorated after implantation of an acoustic nerve device. Although a hypoplastic pituitary gland with interrupted stalk and growth retardation with genital abnormalities were present, his thyroid panel and cortisol levels were normal. Growth hormone (GH) deficiency was confirmed by ornithine test where peak GH reached 0.20 μIU/ml (normal stimulated value > 20 μIU/ml) and by glucagon-propranolol test where peak GH reached 0.0082 μIU/ml (normal stimulated value > 20 μIU/ml). Also, his insulin-like growth factor 1 (IGF1) was at the lower normal value for age (24.6 ng/ml; normal values, 16–215 ng/ml). Therefore, he was started on GH treatment at the age of 4 years.

Due to the presence of a micropenis and undescended testes, he was treated with human chorionic gonadotrophin (HCG). As a result, his penis doubled (length, 2.5 cm) and his testes were in the scrotum. Note that his karyotype was normal male. He received his first testosterone course (100 mg/m^2^; four intramuscular injections at 2-week intervals) at the age of 1 year and 3 months leading to a slight increase in his bone age (2 years and 3 months) as well as penile length (3.5 cm).

At the age of 4 years and 6 months, he received a second course of testosterone which was stopped after two injections because of sudden increase in height (11 cm in 3 months) and bone age (9 years). Random luteinizing hormone and follicle stimulating hormone were low (levels not available) and his testes were in the prepubertal range (2 × 2 cm) ruling out the possibility of a central precocious puberty and this manifestation was related to the second course of testosterone treatment. GH treatment was stopped to slow down the growth velocity and resumed after 3 years during which he was lost to follow-up, and at IGF1 level of 60.8 ng/ml (88–474 ng/ml).

**Fig. 2:**
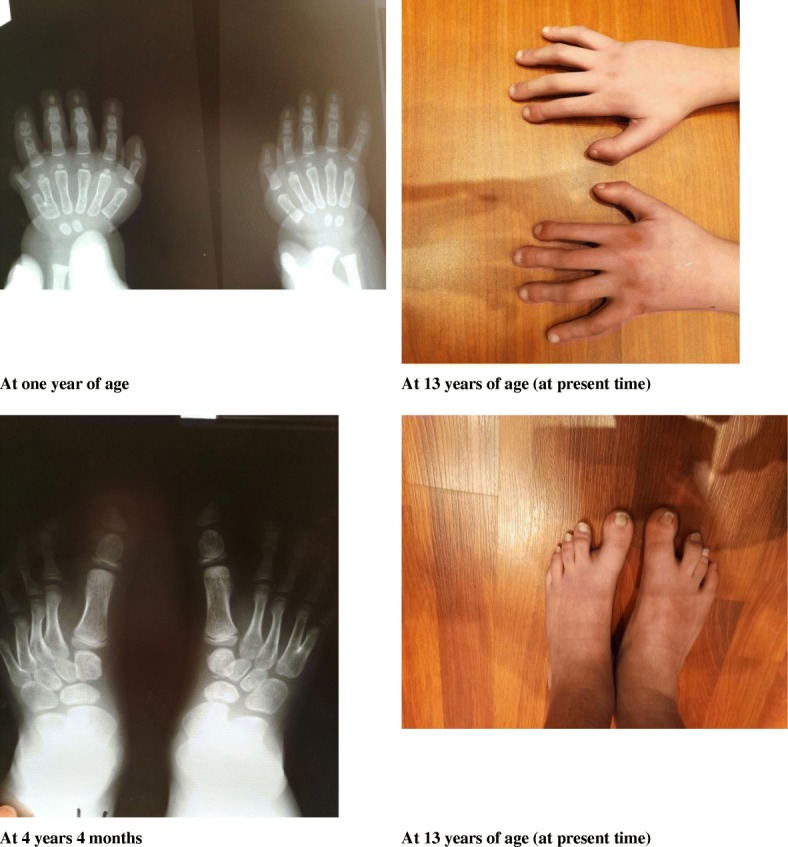
Evolution of the skeletal malformations

**Fig. 3:**
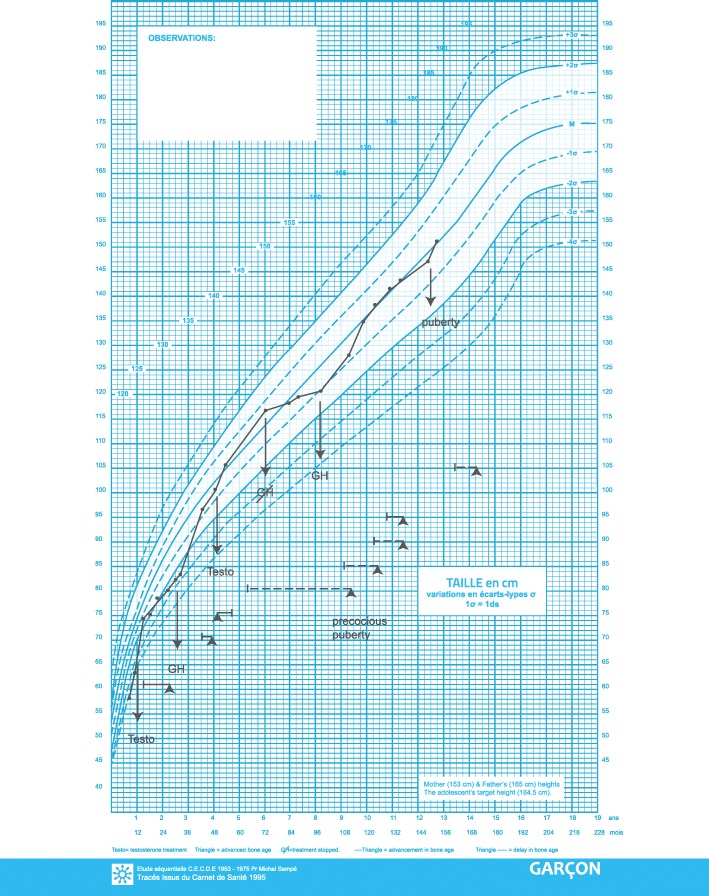
Evolution of the patient’s growth using the French reference curve of height-for-age for boys. *GH* growth hormone

At present, at the age of 13 years, he has a spontaneous puberty (Figs. 2 and 3). His bone age is 13 years and 9 months, he measures 154.5 cm (+ 0.5 standard deviation; SD), he weighs 50 kg (+ 1.5 SD), with a Tanner staging of A2G2. His testes are both in the scrotum and his phallus length is 5 cm.

## Discussion and conclusions

This is a case of a boy with PHS, which is a rare autosomal disease (prevalence < 1%) [11]. The main manifestations of PHS in our patient were hypothalamic hamartoma, brachydactyly, post-axial polydactyly of his right hand, broad great toes with syndactyly, anal stenosis, and a micropenis. However, the choanal atresia was the starting manifestation that led to the clinical investigations.

It was noticed that this case of PHS was not associated with a bifid epiglottis but a narrow orofacial opening. Bifid epiglottis is a congenital malformation defined as a midline cleft of the epiglottis. It is a rare case and Tsurumi *et al.* reported encountering four cases of bifid epiglottis during 10 years [12]. Ondrey *et al*. reported that 15/26 patients with PHS had bifid epiglottis [13]. Also, another study reported that bifid epiglottis occurs in 40% of patients with PHS [14] but none had narrowing of the esophageal opening to our knowledge. In 2016, a case of a 14-year-old girl with PHS was reported to have a bifid epiglottis [15].

An MRI of the brain demonstrated the presence of hypothalamic hamartoma in our patient. Hypothalamic hamartoma is a benign tumor of the hypothalamus and does not need treatment from a tumor perspective. However, other problems are associated with such a condition including treatment-resistant epilepsy, behavioral problems, and endocrine disturbances, most commonly central precocious puberty [16]. This patient was treated by anticonvulsants at the age of 6 months after seizures onset. Otherwise, he was showing a normal neurological development. However, his poor school performance until the age of 10 years was linked to a problem in the electric conductance of the acoustic nerve. The *GLI3* gene is expressed in the otic vesicles and its deletion like in PHS would lead to hearing abnormalities [17]. So, his poor school performance may have been prevented by discovering this medical problem earlier.

It is always challenging to differentiate between syndromic and non-syndromic limb malformations because clinical details in most cases are sparse. It may range from as little as dysplastic nails to pre-axial or post-axial polysyndactyly [18, 19]. The necessary evaluations most of the time are not carried out or neglected because we consider that it is the field of surgeons or because limb malformation is almost never a life-threatening event.

In limb malformations, many syndromes overlap and it is mandatory to go beyond skeletal abnormalities to search for associated manifestations [20]. For example, the association with hypothalamic hamartoma is always a signature of PHS [4]; an association with mental retardation may point to Rubinstein syndrome [16].

Our patient was suffering from growth retardation with genital abnormalities. GH deficiency was confirmed and GH treatment was initiated at the age of 4 years. However, we were concerned about the safety of GH treatment because of the possible stimulating effect of GH on tumor regrowth. *In vitro* and animal experiments suggest that it can raise the risk for hyperplasia and malignancy [21–25]. To the best of our knowledge, no such study is available in the context of hypothalamic hamartoma in humans, but we did find some similarity with studies concerning non-functioning pituitary adenomas (NFPA), a more common tumor, benign in nature with very long stability [26–30]. These studies provide evidence that GH treatment does not appear to increase tumor progression [26–30]. In fact, while on treatment, we did follow our patient with repeated MRI once per year and there was no change in tumor size and no tumor progression.

Regarding genital abnormalities, this patient presented with micropenis and undescended testes. Such findings were also reported in different cases of PHS. Graham *et al.* [31] suggested that micropenis and cryptorchism in male patients with PHS were caused by absent or diminished gonadotropins during fetal development and that hypopituitarism resulted from disruption of normal relationships between the pituitary and the hypothalamus due to hypothalamic hamartoma.

This presentation has the advantage of showing our practical approach to this very rare syndrome and its follow-up in the long term. Being familiar with such cases may allow improvement of our knowledge for better management in the future. Also, our patient presented with tethered cord due to fibrolipoma and an orofacial narrowing, two manifestations that have never been listed as associated with PHS to the best of our knowledge.

## Abbreviations

EEG: Electroencephalogram
GH: Growth hormone
HCG: Human chorionic gonadotrophin
IGF1: Insulin-like growth factor 1
MRI: Magnetic resonance imaging
NFPA: Non-functioning pituitary adenomas
PHS: Pallister–Hall syndrome
SD: Standard deviation
